# A randomized, double-blind, placebo-controlled study assessing the safety and tolerability of regadenoson in subjects with asthma or chronic obstructive pulmonary disease

**DOI:** 10.1007/s12350-012-9547-4

**Published:** 2012-04-07

**Authors:** Bruce M. Prenner, Stan Bukofzer, Sarah Behm, Kathleen Feaheny, Bruce E. McNutt

**Affiliations:** 1 Allergy Associates Medical Group, Inc., 6386 Alvarado Court, Suite 210, San Diego, CA 92120 USA; 2 Astellas Pharma Global Development, Inc., 3 Parkway North, Deerfield, IL USA

**Keywords:** Regadenoson, adenosine, asthma, COPD, MPI, FEV_1_

## Abstract

**Background:**

Adenosine receptor stress agents for myocardial perfusion imaging (MPI) may cause A_2B_ and/or A_3_ receptor-mediated bronchoconstriction, of particular concern to physicians testing patients with asthma or chronic obstructive pulmonary disease (COPD).

**Methods:**

A Phase 4, randomized, double-blind study (NCT00862641) assessed the safety of the selective A_2A_ receptor agonist, regadenoson, compared with placebo in subjects with asthma or COPD who represented likely candidates for MPI.

**Results:**

Overall, 356 and 176 subjects with asthma and 316 and 151 subjects with COPD received regadenoson and placebo, respectively. The percentage of subjects experiencing a >15% decrease in FEV_1_ from baseline to any assessment up to 24 hours post-baseline was not statistically significantly different between the regadenoson and the placebo groups in the asthma or COPD stratum. Dyspnea, the most frequent respiratory adverse event, occurred with higher incidence (*P* < .0001) in the regadenoson group than the placebo group in the asthma (10.7% vs 1.1%) and COPD (18.0% vs 2.6%) strata. No subjects experienced severe bronchoconstriction, although the occurrence of such reactions with adenosine receptor agonists cannot be ruled out, such that caution is advised.

**Conclusions:**

This information may be helpful to physicians selecting a pharmacologic stress agent for MPI in patients with asthma or COPD.

## Introduction

Myocardial perfusion imaging (MPI) is a widely used technique to aid the diagnosis and the evaluation of coronary artery disease. Pharmacologic stress agents are indicated for use in patients who are unable to undergo adequate exercise stress due to physical limitations or medical constraints.^1^ Adenosine, dipyridamole, and regadenoson increase myocardial blood flow by causing coronary vasodilation via stimulation of adenosine A_2A_ receptors.^2^ Pharmacologic stress agents that are non-selective adenosine receptor agonists activate all adenosine receptor subtypes (A_1_, A_2A_, A_2B_, and A_3_) to different extents at recommended clinical doses.^1^ The activation of the A_1_, A_2B_, and A_3_ receptors can elicit a variety of undesirable responses including atrioventricular block (A_1_ receptor), peripheral vasodilation (A_2B_ receptor), and bronchoconstriction (A_2B_ and A_3_ receptors).^1^ The risk of bronchoconstriction is of particular concern for physicians considering pharmacologic stress MPI in patients with asthma or chronic obstructive pulmonary disease (COPD).

As regadenoson has a greater affinity for the A_2A_ receptor and much lower affinity for the other adenosine receptor subtypes at recommended clinical doses^1^ compared with adenosine or dipyridamole, the risk of bronchoconstriction in patients with reactive airways could be lower.^3^,^4^ Two pilot studies have been conducted in subjects with asthma^5^ and COPD.^6^ In the first pilot study, Leaker et al^5^ conducted a randomized, double-blind, placebo-controlled, crossover study of 48 subjects with mild or moderate asthma who demonstrated bronchial reactivity to adenosine monophosphate. The mean ratio of the forced expiratory volume in 1 second (FEV_1_) to the baseline FEV_1_ was higher in the regadenoson group than the placebo group from 10 to 60 minutes after drug administration.^5^ Mean FEV_1_ in the regadenoson group was not statistically different from the placebo group at any of the scheduled assessments up to 120 minutes post-dose. Bronchoconstrictive reactions (defined by Leaker et al as a >15% reduction in FEV_1_ from baseline) were experienced by similar percentages of subjects who received regadenoson (4.3% [2/47]) and placebo (4.2% [2/48]), and were not associated with pulmonary adverse events, serious adverse events, or study termination.^5^ In the second pilot study, Thomas et al^6^ performed a randomized, double-blind, placebo-controlled crossover trial of regadenoson safety in 49 subjects with moderate or severe COPD. No differences were observed between subjects who received regadenoson and those who received placebo across lung function parameters, which included FEV_1_, forced vital capacity (FVC), respiratory rate, pulmonary examinations, and oxygen saturation.^6^ There were no statistically significant differences in the mean FEV_1_ between the regadenoson group and the placebo group at any of the scheduled assessments up to 120 minutes post-dose. Bronchoconstrictive reactions (defined by Thomas et al as a >15% reduction in FEV_1_ from baseline) occurred in 12.2% (6/49) of subjects who received regadenoson and 6.1% (3/49) of subjects who received placebo. These changes were not associated with pulmonary adverse events (other than throat tightness in one subject who received regadenoson), changes in vital signs, pulmonary examination, or oxygen saturation, and no treatment was required.^6^

This study was conducted to further investigate the safety of regadenoson for use in patients with asthma or COPD.

## Materials and Methods

A Phase 4, multicenter, randomized, double-blind, placebo-controlled study to assess the safety and the tolerability of regadenoson in subjects with asthma and/or COPD who represent those likely to be referred for MPI studies was conducted (NCT00862641). The study was not designed to assess the efficacy of regadenoson for MPI in subjects with asthma or COPD; thus, subjects did not undergo imaging studies following administration of study drug.

### Inclusion and Exclusion Criteria

Subjects with asthma and/or COPD were eligible for enrollment if they were ≥18 years of age. In order to represent patients who were likely candidates for MPI procedures, subjects were to have ongoing coronary artery disease, or have at least two of the following risk factors for coronary artery disease: type 2 diabetes, hypertension, hypercholesterolemia, current or history of cigarette smoking (minimum 10 pack years exposure), or obesity (body mass index [BMI] >30 kg/m^2^). Asthmatic subjects were to have reasonably well-controlled asthma (as demonstrated by an FEV_1_ ≥60% predicted) with the frequency and severity of their symptoms having remained unchanged within 30 days prior to study drug administration. COPD was defined by an FEV_1_/FVC ratio <0.70.

Subjects were to be excluded if they had participated in another drug study or received an investigational drug within 30 days prior to screening. Subjects were not included if they had a history of additional clinically significant illnesses, medical conditions, or laboratory abnormalities within 2 weeks prior to screening that, in the investigator’s opinion, would hinder study procedures or confuse the interpretation of data. Furthermore, subjects with a history of second- or third-degree heart block or sinus node dysfunction (unless the subject had a functioning pacemaker), symptomatic hypotension, or who had a respiratory infection within 2 weeks or surgery within 3 months prior to randomization, were excluded.

Subjects were required to have a stable medication regime for a minimum of 30 days prior to study drug administration, without an exacerbation of their asthma or COPD during this same period. Subjects must not have started on a course of oral or injectable corticosteroids, inhaled steroid combination with a long-acting β_2_ agonist (oral or inhaled) or anticholinergic, or had undergone a change in dose of such medications ≤30 days prior to study drug administration. Subjects must not have started or changed the dose of any leukotriene antagonists, mast cell stabilizers (cromones), or 5-lipoxygenase antagonists ≤7 days prior to study drug administration. Subjects were not to have an allergy or intolerance to aminophylline or regadenoson or any of its excipients.

Female subjects who were pregnant or had a positive pregnancy test within 24 hours prior to randomization, lactating, or of childbearing potential and refused to use a medically acceptable form of contraception until the follow-up visit was complete were also excluded.

Written informed consent and privacy language was obtained from all subjects or legally authorized representatives prior to any study-related procedures, in accordance with the respective Instructional Review Boards or Ethics Committees and appropriate Federal regulations.

### Concomitant Medications

Subjects had to abstain from any intake of methylxanthine-containing foods and beverages within 12 hours prior to study drug administration until the follow-up visit. In addition, all subjects had to abstain from theophylline for 12 hours prior to the Day 1 visit. When possible, dipyridamole was withheld for at least 2 days prior to study drug administration. Subjects were not allowed to alter their concomitant medications from study drug administration until the follow-up visit.

### Randomization and Blinding

The study was a double-blind, randomized, parallel group study stratified by the underlying condition of asthma or COPD. Subjects who met the inclusion and exclusion criteria were randomized into the study using a 2:1 (regadenoson/placebo) computer-generated randomization schedule by stratum (asthma or COPD). The randomization schedule was generated by Astellas. Study drug assignment was to be revealed only for reasons relating to the safety of the subject, when knowledge of the assigned drug was needed to make critical therapeutic decisions.

### Drug Administration

Subjects were to abstain from eating and drinking for 30 minutes prior to and 30 minutes post-study drug administration, and were to abstain from smoking for 3 hours prior and 8 hours post-study drug administration. Subjects received either a 0.4 mg/5 mL (0.08 mg/mL) intravenous bolus injection of regadenoson or matching placebo, administered over approximately 10 seconds, followed by a 5-mL 0.9% saline flush. Subjects were placed in either a reclining or a supine position prior to dosing and remained in that position for the first 2 hours (except during spirometry measurements). The protocol did not require physicians to administer short-acting β_2_ agonists (bronchodilators) prior to the administration of regadenoson, although subjects were instructed to continue their normal prescribed respiratory medications prior to study drug administration. After study drug administration, short-acting β_2_ agonists were used for mild-to-moderate respiratory or cardiac symptoms, followed by intravenous aminophylline if the short-acting β_2_ agonist did not successfully treat the symptoms. Aminophylline was used as the primary treatment for any severe and/or persistent respiratory or cardiac symptoms.

### Safety Assessments

The majority of adverse events for regadenoson occur during the 2-hour period after administration; thus, the primary safety objective selected for this study was to determine the percentage of subjects with a >15% decrease in FEV_1_ from baseline to the 2-hour post-baseline assessment. A >15% decline in FEV_1_ from baseline was chosen as a clinically meaningful indication of airway obstruction. Used in the diagnosis of asthma, a >15% decrease in FEV_1_ in an exercise challenge test is diagnostic of exercise-induced bronchoconstriction.^7^,^8^ Although, changes in respiratory function may be expected to occur before 2 hours post-baseline, subjects with a >15% reduction in FEV_1_ from baseline to this assessment would represent those with significant, persistent respiratory function changes. FEV_1_ measurements were obtained at 5 minutes, 15 minutes, 1 hour, 2 hours, 8 hours, and 24 hours (follow-up visit) post-study drug administration. The effect of baseline asthma and COPD severity on FEV_1_ decreases was also investigated. Asthma severity was assessed jointly by the Astellas Medical Director and an external asthma expert (Dr Bruce M. Prenner, Allergy Associates Medical Group, Inc.) using a modified version of the National Heart, Lung, and Blood Institute (NHLBI) Expert Panel Report 3 stepwise approach for managing asthma, based on the type and dose of asthma medication (Table 1).^9^ In the event of a difference of opinion between the parties, the classification of the asthma expert was utilized. COPD severity was assessed by the investigator at each study site using the Global Initiative for Chronic Obstructive Lung Disease (GOLD) criteria, based on lung function parameters (Table 1).^10^ Oxygen saturation was measured continuously by pulse oximetry from baseline through the 2-hour post-baseline assessment; measurements at 1, 2, 5, 10, 15, and 30 minutes, and 1 and 2 hours post-baseline were analyzed.

**Table 1:** Asthma and COPD severity criteria^9^,^10^

*Asthma*
Modified version of National Heart, Lung, and Blood Institute criteria^9^
Step 1: Inhaled short-acting β_2_ agonist when needed
Step 2: Low-dose inhaled corticosteroid OR cromolyn, leukotriene receptor antagonist, nedocromil, or theophylline
Step 3: Low-dose inhaled corticosteroid and long-acting β_2_ agonist OR medium-dose inhaled corticosteroid OR low-dose inhaled corticosteroid, and either leukotriene receptor antagonist, theophylline, or zileuton
Step 4: Medium-dose inhaled corticosteroid and long-acting β_2_ agonist, OR medium-dose inhaled corticosteroid and either leukotriene receptor antagonist, theophylline, or zileuton
Step 5: High-dose inhaled corticosteroid and long-acting β_2_ agonist
Step 6: High-dose inhaled corticosteroid and long-acting β_2_ agonist and oral steroids
*COPD*
GOLD criteria^10^
Stage I (mild): FEV_1_/FVC <0.70, FEV_1_ ≥80% predicted
Stage II (moderate): FEV_1_/FVC <0.70, 50%≤FEV_1_<80% predicted
Stage III (severe): FEV_1_/FVC <0.70, 30%≤FEV_1_<50% predicted
Stage IV (very severe): FEV_1_/FVC <0.70, FEV_1_ <30% predicted or FEV_1_ <50% predicted plus chronic respiratory failure

*COPD*, Chronic obstructive pulmonary disease; *FEV*_*1*_ *,* forced expiratory volume in 1 second; *FVC*, forced vital capacity.

Other safety assessments included the incidence of selected respiratory adverse events (defined prior to unblinding as dyspnea, wheezing, obstructive airways disorder, exertional dyspnea, and tachypnea [MedDRA version 11.1 preferred terms]) up to 24 hours post-regadenoson administration, and the use of short-acting bronchodilators in response to these events. Vital signs (heart rate and blood pressure) and 12-lead electrocardiograms (ECGs) were monitored at selected intervals up to 24 hours post-study drug administration.

### Statistical Analyses

The protocol planned for the enrollment of 450 subjects in each of the asthma and COPD strata (with 300 subjects receiving regadenoson in each stratum). The analysis population consisted of all randomized subjects who received any amount of study drug.

Descriptive statistics were calculated for continuous variables, and frequencies and percentages were displayed for categorical data. All statistical comparisons were made using 2-sided tests at the α = 0.05 significance level unless stated otherwise. All null hypotheses were of no treatment difference and all alternative hypotheses were 2-sided. Demographics and other baseline characteristics were summarized using descriptive statistics; statistical comparisons between the treatment groups were summarized. One-way analysis of variance (ANOVA) was used for continuous variables and Fisher’s exact test or chi-square for the discrete variables.

### Protocol Amendments

Notable protocol amendments made after the trial had commenced included the modification of the primary endpoint to be pulmonary function as measured by the proportion of subjects with a >15% decrease in FEV_1_ from baseline to the 2-hour post-baseline assessment, rather than the assessment of selected respiratory adverse events (“protocol amendment 3”; made after 692 subjects had been enrolled). Also under protocol amendment 3, an additional inclusion criterion was added for COPD subjects (FEV_1_/FVC <70%), and two additional inclusion criteria were added for asthma subjects (FEV_1_ ≥60% predicted and the frequency and severity of their symptoms having remained unchanged within 30 days prior to study drug administration). The addition of current or history of cigarette smoking (minimum 10 pack years exposure) as a risk factor for coronary artery disease (“protocol amendment 2”) was added after 81 subjects had been enrolled.

## Results

### Subject Disposition and Baseline Characteristics

**Figure 1:**
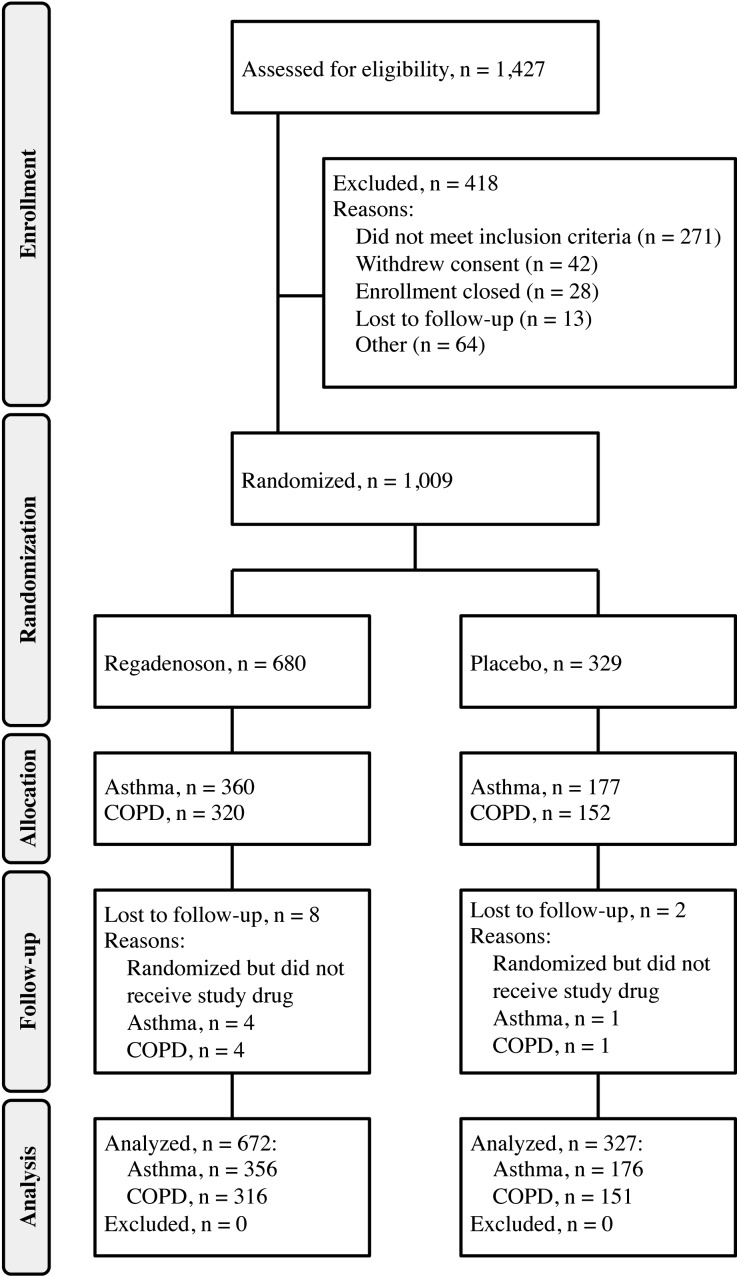
Subject disposition flow diagrams for the asthma (**A**) and COPD (**B**) disease strata

In total, 1,009 subjects were randomized from 48 study centers in the United States. Study drug was administered to 999 subjects, of which 356 and 176 subjects with asthma and 316 and 151 subjects with COPD received regadenoson and placebo, respectively (Figure 1). Five subjects in each disease stratum (four who were randomized to receive regadenoson and one who was randomized to receive placebo) were randomized but did not receive study drug.

Baseline characteristics were generally similar between subjects who received regadenoson and subjects who received placebo in the two disease strata, although subjects with COPD who received regadenoson had a statistically significantly lower mean body weight and BMI than subjects who received placebo (Table 2). Baseline asthma or COPD severity^9^,^10^ was similar between subjects who received regadenoson or placebo, although a statistically significant difference (*P* = .0095) was observed for the distribution of asthma subjects due to the Step 4 asthma category, which comprised a greater number of subjects who received regadenoson than placebo. These differences are likely an artifact of the randomization (which was not stratified by asthma/COPD severity).

**Table 2:** Baseline subject demographics, including medical history and risk factors for coronary artery disease

Parameter	Asthma stratum		COPD stratum	
	Placebo N = 176	Regadenoson N = 356	Placebo N = 151	Regadenoson N = 316
Female, n (%)	115 (65.3)	245 (68.8)	67 (44.4)	132 (41.8)
Mean age (years) (SD)	52.3 (11.6)	52.2 (11.8)	60.0 (10.4)	60.4 (10.7)
Age ≥65 years, n (%)	28 (15.9)	52 (14.6)	56 (37.1)	127 (40.2)
Mean weight (kg) (SD)	99.5 (23.7)	97.2 (21.6)	94.2 (23.8)*	89.5 (20.3)*
Mean BMI (kg/m^2^) (SD)	35.5 (8.0)	35.2 (7.4)	32.4 (7.3)**	30.9 (6.5)**
Race, n (%)				
White	128 (72.7)	283 (79.5)	136 (90.1)	284 (89.9)
Black/African American	45 (25.6)	61 (17.1)	13 (8.6)	22 (7.0)
Other	3 (1.7)	12 (3.4)	2 (1.3)	10 (3.2)
Medical history, n (%)				
COPD	4 (2.3)	10 (2.8)	151 (100)	316 (100)
Asthma	176 (100)	356 (100)	8 (5.3)	17 (5.4)
Current CAD	21 (11.9)	34 (9.6)	35 (23.2)	63 (19.9)
Ongoing CAD risk factors				
Hypercholesterolemia	120 (68.2)	243 (68.3)	104 (68.9)	215 (68.0)
Hypertension	134 (76.1)	250 (70.2)	115 (76.2)	237 (75.0)
Obesity (BMI >30 kg/m^2^)	132 (75.0)	282 (79.2)	94 (62.3)	163 (51.6)
Type 2 diabetes	48 (27.3)	98 (27.5)	51 (33.8)	77 (24.4)
Current smoker, or history of smoking^a^, n (%)	57 (32.4)	122 (34.3)	130 (86.1)	268 (84.8)

*BMI*, body mass index; *CAD*, coronary artery disease; *COPD*, chronic obstructive pulmonary disease; *SD*, standard deviation.

*P = .0276, **P = .0301.

^a^Smoking defined as ≥10 pack years.

### Safety Assessments

The percentage of subjects with a >15% decrease in FEV_1_ from baseline to the 2-hour post-baseline assessment was not statistically significantly different between the regadenoson group and the placebo group in either the asthma stratum (1.1% [4/351] vs 2.9% [5/174], respectively) or the COPD stratum (4.2% [13/313] vs 5.4% [8/147], respectively) (Table 3). Furthermore, there were no significant differences in the percentage of subjects with a >15% decrease in FEV_1_ from baseline to any assessment up to the follow-up visit, 24 hours post-baseline (Table 3). The percentage of subjects with a >15% decrease in change in FEV_1_ was not affected by baseline disease severity in either the asthma or COPD stratum (Table 4). The largest individual absolute decreases in FEV_1_ in the regadenoson group were 1.1 L (33.1%) and 0.79 L (30.7%) in the asthma and COPD strata, respectively; the lowest measurements were both obtained at the 15-minute post-dose assessment (Table 5). The largest individual percentage decreases in FEV_1_ in the regadenoson group were 34.4% (0.64 L; 15 minutes post-dose) and 44.6% (0.78 L; 5 minutes post-dose) in the asthma and COPD stratum, respectively (Table 5). The largest individual FEV_1_ decreases were generally slightly lower in the placebo group, with the lowest individual measurements obtained at a variety of post-dose assessments from 5 minutes to 2 hours post-dose (Table 5).

**Table 3:** Subjects with a >15% decrease in FEV_1_ from baseline to each post-baseline assessment

Visit	Placebo n/N (%)	Regadenoson n/N (%)	Difference (regadenoson − placebo) [95% CI]^a^	*P* value^b^
Asthma stratum				
Minute 5	1/161 (0.6)	5/322 (1.6)	0.9% [−0.9%, 2.8%]	.3941
Minute 15	4/175 (2.3)	2/355 (0.6)	−1.8% [−4.1%, 0.6%]	.0685
Hour 1	1/86 (1.2)	0/175 (0.0)	−1.1% [−3.2%, 1.0%]	.2032
Hour 2^c^	5/174 (2.9)	4/351 (1.1)	−1.8% [−4.5%, 1.0%]	.1451
Hour 8^d^	2/23 (8.7)	2/40 (5.0)	−3.9% [−17.5%, 9.7%]	.4151
Follow-up^e^	2/83 (2.4)	3/173 (1.7)	−0.7% [−4.4%, 3.0%]	.7573
At any visit^f^	12/176 (6.8)	13/356 (3.7)	−3.2% [−7.5%, 1.0%]	.1004
COPD stratum				
Minute 5	4/132 (3.0)	10/272 (3.7)	0.7% [−3.0%, 4.4%]	.7315
Minute 15	5/150 (3.3)	9/314 (2.9)	−0.4% [−3.9%, 3.1%]	.8134
Hour 1	1/13 (7.7)	2/30 (6.7)	−8.7% [−25.8%, 8.4%]	.1138
Hour 2^c^	8/147 (5.4)	13/313 (4.2)	−1.2% [−5.4%, 3.1%]	.5790
Hour 8^d^	4/14 (28.6)	5/39 (12.8)	−20.7% [−48.4%, 7.1%]	.0971
Follow-up^e^	2/27 (7.4)	4/58 (6.9)	−2.0% [−15.4%, 11.3%]	.7237
At any visit^f^	13/151 (8.6)	31/316 (9.8)	1.2% [−4.3%, 6.7%]	.6675

*CI*, confidence interval; *COPD*, chronic obstructive pulmonary disease; *FEV*_*1*_ *,* forced expiratory volume in 1 second.

^a^Treatment effect and 95% confidence intervals were calculated using weighted averages where the weights adjusted for the number of subjects in each investigative site. These weights reflect their relative contribution to the Mantel-Haenszel statistic.

^b^Cochran-Mantel-Haenszel test stratified by investigative site. Sites with <15 subjects were pooled.

^c^Primary safety outcome.

^d^Only includes subjects with FEV_1_ Hour 2 results not within 15% of baseline results or who were symptomatic at Hour 2.

^e^Follow-up: 24 hours post-drug administration; includes subjects with FEV_1_ Hour 2 results not within 15% of baseline results or who were symptomatic at Hour 2 and all subjects randomized under protocol amendment 3.

^f^FEV_1_ post-baseline assessments were performed at the Minute 5, Minute 15, Hour 1, Hour 2, Hour 8, and follow-up visit.

**Table 4:** Subjects with a >15% decrease in FEV_1_ from baseline to the 2-hour post-baseline assessment according to baseline disease severity

	Placebo n/N (%)	Regadenoson n/N (%)	*P* value^a^
Asthma stratum^b,c^			
Step 1	0/47 (0)	2/107 (1.9)	.3368
Step 2	0/27 (0)	1/33 (3.0)	.2207
Step 3	3/31 (9.7)	1/49 (2.0)	.1673
Step 4	0/23 (0)	0/84 (0)	–
Step 5	1/25 (4.0)	0/44 (0)	.1904
Step 6	1/2 (50.0)	–	–
Unable to classify^d^	0/19 (0)	0/34 (0)	–
COPD stratum^e^			
Stage I	1/20 (5.0)	0/43 (0)	–
Stage II	1/72 (1.4)	9/148 (6.1)	.1353
Stage III	2/28 (7.1)	2/60 (3.3)	.6877
Stage IV	1/2 (50.0)	0/4 (0)	.3173
Not calculated^f^	3/25 (12.0)	2/58 (3.4)	.1749

*COPD*, Chronic obstructive pulmonary disease; *FEV*_*1*_ *,* forced expiratory volume in 1 second.

^a^Cochran-Mantel-Haenszel test stratified by investigative site. Sites with <15 subjects were pooled.

^b^Asthma severity assessed using a modified version of the National Heart, Lung, and Blood Institute (NHLBI) Expert Panel Report 3 (Table 1).^9^

^c^The distribution of baseline asthma severity categories was significantly different between the regadenoson and placebo groups (*P* = .0095) because of a greater percentage of Step 4 subjects in the regadenoson group than in the placebo group.

^d^Subjects were not classified if no prior medications reported.

^e^COPD severity assessed using the Global Initiative for Chronic Obstructive Lung Disease (GOLD) criteria (Table 1).^10^

^f^Subjects enrolled prior to protocol amendment 2 and who did not complete test for classifying severity and/or subjects who did not meet GOLD criteria for severity.

**Table 5:** Greatest individual decreases in oxygen saturation and FEV_1_

Parameter	Disease stratum Treatment group	Decrease	Baseline measurement	Lowest post-baseline measurement	Assessment at which lowest measurement recorded
Oxygen saturation, greatest % decrease	Asthma stratum				
	Placebo	15.2%	98.7%	83.5%	Minute 5
	Regadenoson	12.4%	97.9%	85.5%	Minute 30
	COPD stratum				
	Placebo	14.4%	95.2%	80.8%	Hours 3–7
	Regadenoson	12.7%	98.2%	85.5%	Minute 30
FEV_1_, greatest % decrease	Asthma stratum				
	Placebo	0.74 L (32.9%)	2.25 L	1.51 L	Hour 2
	Regadenoson	0.64 L (34.4%)	1.86 L	1.22 L	Minute 15
	COPD stratum				
	Placebo	0.50 L (29.9%)	1.67 L	1.17 L	Hour 2
	Regadenoson	0.78 L (44.6%)	1.75 L	0.97 L	Minute 5
FEV_1_, greatest absolute decrease	Asthma stratum				
	Placebo	1.06 L (32.3%)	3.28 L	2.22 L	Minute 15
	Regadenoson	1.11 L (33.1%)	3.35 L	2.24 L	Minute 15
	COPD stratum				
	Placebo	0.55 L (28.8%)	1.91 L	1.36 L	Minute 5
	Regadenoson	0.79 L (30.7%)	2.57 L	1.78 L	Minute 15

*COPD*, Chronic obstructive pulmonary disease; *FEV*_*1*_, forced expiratory volume in 1 second.

Decreases in FEV_1_ and FVC from baseline to 2 hours post-dose for the regadenoson group were similar to, or less than the placebo group (Table 6).

**Table 6:** Respiratory parameters at baseline and change at 2 hours post-baseline

	Placebo		Regadenoson		*P* value^a^
	n	Mean ± SD	n	Mean ± SD	
*Asthma stratum*					
FEV_1_ (L)					
Baseline	176	2.41 ± 0.689	356	2.35 ± 0.623	
Change at Hour 2	174	−0.05 ± 0.160	351	−0.01 ± 0.129	.0029**
FVC (L)					
Baseline	176	3.22 ± 0.883	356	3.16 ± 0.829	
Change at Hour 2	174	−0.06 ± 0.208	351	−0.03 ± 0.168	.0789
FEV_1_/FVC ratio					
Baseline	176	74.88 ± 7.510	356	74.59 ± 7.084	
Change at Hour 2	174	0.16 ± 3.329	351	0.52 ± 2.562	.2087
*COPD stratum*					
FEV_1_ (L)					
Baseline	151	1.70 ± 0.630	316	1.70 ± 0.655	
Change at Hour 2	147	−0.01 ± 0.152	313	−0.00 ± 0.159	.6189
FVC (L)					
Baseline	151	2.75 ± 0.833	316	2.83 ± 0.899	
Change at Hour 2	147	−0.04 ± 0.230	313	0.00 ± 0.239	.0258*
FEV_1_/FVC ratio					
Baseline	151	61.95 ± 12.684	316	60.00 ± 13.191	
Change at Hour 2	147	0.31 ± 3.177	313	−0.30 ± 3.571	.0289*

*COPD*, Chronic obstructive pulmonary disease; *FVC*, forced vital capacity; *FEV*_*1*_, forced expiratory volume in 1 second; *SD*, standard deviation.

*^,^**Statistical significance at level of *P* = .05 and *P* = .01, respectively.

^a^ *F* test from ANCOVA with treatment and investigator site as main effects and baseline value as a covariate in the model.

Decreases in oxygen saturation are a severe manifestation of acute pulmonary decomposition. Changes in oxygen saturation from baseline to any post-baseline assessment did not demonstrate statistically significant decreases in the regadenoson group compared with the placebo group in either the asthma or COPD stratum, with the exception of the 30-minute post-baseline measurement in the COPD stratum (−0.8% in the regadenoson group vs 0% in the placebo group). The greatest individual decreases in oxygen saturation in the regadenoson group were −12.4% (97.9% to 85.5%) and −12.7% (98.2% to 85.5%) in the asthma and COPD strata, respectively; both lowest measurements were recorded at the 30-minute post-dose assessment (Table 5). The subjects with the greatest individual decreases in oxygen saturation and the aforementioned subjects with the greatest individual decreases in FEV_1_ were not the same subjects. The greatest individual decreases in oxygen saturation in the regadenoson group were less than the corresponding decreases observed in the placebo group (−15.2%, 5 minutes post-dose and −14.4%, 3 to 7 hours post-dose for the asthma and COPD strata, respectively) (Table 5).

Two subjects in the placebo group (one with COPD and one with asthma) and two subjects in the regadenoson group (both with COPD) had both a decrease in FEV_1_ >15% from baseline and a potentially meaningful decrease in oxygen saturation (to <92% of baseline level). One of the subjects who received regadenoson (COPD stratum) had a >15% FEV_1_ decrease and oxygen saturation drop at 15 minutes and 2 hours post-baseline whereas all the other concurrent falls were at 2 hours post-baseline.

Of the identified selected respiratory adverse events (defined prior to unblinding as dyspnea, wheezing, obstructive airways disorder, exertional dyspnea, and tachypnea), dyspnea occurred most frequently, with a higher incidence (*P* < .0001) in the regadenoson group than the placebo group in both the asthma (10.7% [38/356] vs 1.1% [2/176]) and COPD strata (18.0% [57/316] vs 2.6% [4/151]). In the asthma stratum, wheezing occurred in 3.1% (11/356) of the regadenoson group and 1.1% (2/176) of the placebo group, and obstructive airways disorder (reported as airway constriction) was reported in one regadenoson group subject. In the COPD stratum, all other selected respiratory adverse events occurred in <1% of subjects. In subjects with a >15% decrease in FEV_1_ from baseline to the 2-hour post-baseline assessment, no subjects who received placebo in either disease stratum or any subject in the asthma stratum reported a selected respiratory adverse event up to 1 day post-study drug administration. In the COPD stratum, two subjects with a >15% decrease in FEV_1_ from baseline to the 2-hour post-baseline assessment who received regadenoson reported dyspnea within 2 hours of study drug administration and two subjects reported dyspnea within 1 day of study drug administration. One subject in the COPD stratum with a >15% decrease in FEV_1_ from baseline to the 2-hour post-baseline assessment who received regadenoson reported wheezing within 1 day of study drug administration. There were no significant differences between the regadenoson and the placebo groups (for either disease stratum) in the number of subjects who were using short-acting β_2_ agonists at the time of these selected respiratory adverse events (Table 7). It should be noted that although the protocol did not require physicians to administer short-acting β_2_ agonists prior to the administration of regadenoson, subjects may have been receiving these agents as part of their routine care.

**Table 7:** Short-acting bronchodilator use at the time of selected symptomatic respiratory adverse events

Parameter n (%)	Asthma stratum		COPD stratum	
	Placebo N = 176	Regadenoson N = 356	Placebo N = 151	Regadenoson N = 316
*Within 2 hours of study drug administration*				
Subjects who experienced a respiratory symptomatic adverse event of interest^a^	3 (1.7)	42 (11.8)	5 (3.3)	58 (18.4)
Subjects who used a short-acting bronchodilator^b^	3 (1.7)	3 (0.8)	5 (3.3)	12 (3.8)
Subjects using a short-acting bronchodilator at time of selected adverse event^c^	1 (0.6)	1 (0.3)	2 (1.3)	2 (0.6)
*Within 24 hours of study drug administration*				
Subjects who experienced a respiratory symptomatic adverse event of interest^a^	4 (2.3)	46 (12.9)	6 (4.0)	60 (19.0)
Subjects who used a short-acting bronchodilator^b^	94 (53.4)	168 (47.2)	65 (43.0)	141 (44.6)
Subjects using a short-acting bronchodilator at time of selected adverse event^c^	2 (1.1)	5 (1.4)	2 (1.3)	5 (1.6)

*COPD*, chronic obstructive pulmonary disease.

^a^Dyspnea, wheezing, obstructive airways disorder, exertional dyspnea, and tachypnea (MedDRA version 11.1 preferred terms).

^b^Short-acting bronchodilators defined as medications coded to drugs for obstructive airway diseases.

^c^Use of a short-acting bronchodilator between the start and stop time of the specified respiratory adverse event of interest for each subject.

Adverse events were experienced by 235/356 (66.0%) subjects who received regadenoson and 55/176 (31.3%) subjects who received placebo in the asthma stratum, and 193/316 (61.1%) subjects who received regadenoson and 38/151 (25.2%) subjects who received placebo in the COPD stratum. Adverse events that occurred with an incidence rate of >10% among subjects who received regadenoson were headache (97 subjects, 27.2%), dizziness (69, 19.4%), chest discomfort (44, 12.4%), nausea (43, 12.1%), and dyspnea (38, 10.7%) in the asthma stratum (N = 356), and headache (62, 19.6%), dyspnea (57, 18.0%), dizziness (45, 14.2%), flushing (37, 11.7%), and chest discomfort (35, 11.1%) in the COPD stratum (N = 316); all these events were statistically significantly more frequent in subjects who received regadenoson than placebo. One serious adverse event was reported during the study in an asthmatic subject who received regadenoson (complete atrioventricular block; subject recovered). This event was considered by the investigator as probably related to regadenoson. Three serious adverse events were reported during the study in COPD subjects who received regadenoson; bradycardia was deemed probably related, ECG change (T wave abnormality with possible anterior ischemia) was deemed possibly related, and nephrolithiasis was not considered to be related to regadenoson by the investigators. Two further serious adverse events (rectal hemorrhage and amyotrophic lateral sclerosis) were experienced by subjects who received regadenoson after the follow-up visit, neither of which was considered to be related to regadenoson.

Two regadenoson subjects in the COPD stratum received aminophylline for treatment of adverse events; one subject experienced bradycardia, which was treated with aminophylline 75 mg (intravenous), and one subject experienced dyspnea, which was treated with aminophylline 50 mg (intravenous).

The percentage of subjects with a heart rate >100 bpm at any assessment up to 1 hour post-study drug administration was greater in the regadenoson group than in the placebo group in both the asthma stratum (16.4% [58/354] vs 0.6% [1/173], respectively) and the COPD stratum (11.1% [35/315] vs 0.7% [1/149], respectively). Furthermore, from examination of ECGs, mean increases in heart rate from baseline to 5 minutes post-dose were greater in subjects who received regadenoson than placebo in the asthma stratum (17.2 vs 0.3 bpm, respectively) and the COPD stratum (14.5 vs −0.3 bpm, respectively). The differences between the regadenoson and the placebo groups are consistent with the cardiovascular effects expected for regadenoson,^11^ and lessened at each post-baseline assessment in both disease strata. At any of the intervals assessed, the percentage of subjects with an ECG abnormality was similar in the regadenoson and the placebo groups for both disease strata.

Systolic blood pressure of ≥180 mm Hg with at least a 20 mm Hg increase from baseline at any assessment up to 1 hour post-study drug administration was also observed more frequently in subjects in the COPD stratum who received regadenoson than those who received placebo (2.8% [9/316] vs 0.7% [1/151], respectively). No other significant differences were observed between the regadenoson and the placebo groups with regard to heart rate or blood pressure.

## Discussion

Regadenoson was not statistically significantly different from placebo with respect to the percentage of subjects experiencing a >15% decrease in FEV_1_ from baseline to 2 hours post-baseline, or at any assessment up to the follow-up visit (24 hours post-baseline) in either the asthma or the COPD stratum. The number of subjects with a concurrent >15% decrease in FEV_1_ and drop in oxygen saturation to <92% of the baseline value was the same in the regadenoson and placebo groups (two subjects in each). The change in FEV_1_ was not affected by baseline disease severity in either the asthma or the COPD stratum.

The asthma and the COPD severity classification criteria used in this study are clinical practice guidelines intended to guide patient management.^9^,^10^ Neither are validated for categorization of disease severity. The GOLD COPD guidelines include categorization of severity based on post-bronchodilator FEV_1_ and the NHLBI asthma criteria are based on the type and dose of asthma medication. Asthma is a dynamic disease, with patients experiencing fluctuations in symptom frequency and severity, such that the treatment that a patient is receiving (on which the NHLBI asthma criteria are based) may, and will, vary over time. In order to optimally assess asthma severity, interpretation of the patient’s treatment history in the period preceding the study is needed. In this study, that history involved the 30 days preceding administration of study drug. Furthermore, in accordance with the inclusion/exclusion criteria of the study, subjects were not randomized if they changed their asthma treatment regimen or experienced an exacerbation due to their asthma. It is, therefore, thought that the asthma and the COPD criteria employed in this study are a realistic proxy of disease severity.

Although the subjects in this study had a wide range of asthma and COPD severity, their underlying disease was required by the inclusion/exclusion criteria of the study to be stable. Thus, the results of this study cannot be extrapolated for subjects who have acute exacerbation of their underlying illness necessitating a change in their treatment regimen. Nonetheless, we believe that this study achieved the aim of evaluating the safety of regadenoson in subjects with a wide range of severity of asthma or COPD. Furthermore, consistent with accepted guidelines, the respiratory condition of patients with asthma or COPD should be as stable as possible prior to MPI with any pharmacologic stress agent.^3^

The overall incidence of selected respiratory adverse events was statistically significantly higher in the regadenoson group compared with placebo in both the asthma and the COPD strata, but this did not result in a greater use of short-acting β_2_ agonists at the time of these selected respiratory adverse events. The types of adverse events reported in this study were similar to those observed in other studies of regadenoson.^12^-^14^

Severe bronchoconstrictive reactions have been reported following adenosine and dipyridamole, and although rare, are of serious concern to clinicians.^15^,^16^ In this study, 672 subjects received regadenoson. A sample size of 300 receiving regadenoson in each disease stratum would be able to detect an adverse event as rare as 54 per 10,000 with approximately 80% probability, and 76 per 10,000 with approximately 90% probability. Although no such severe reactions occurred during this study, this does not exclude the possibility of these occurring when adenosine receptor agonists are used in the clinical setting. Consistent with the caution advised in the regadenoson prescribing information, physicians should have the appropriate medications and resuscitation equipment available during regadenoson stress in patients with asthma or COPD in the event of bronchoconstriction.^11^

The number of subjects in this study with known coronary artery disease was 15.3% (N = 153) compared with 77.0% (N = 1,441) in the pivotal ADVANCE MPI (ADenosine Versus regAdeNoson Comparative Evaluation for Myocardial Perfusion Imaging) trials.^12^,^14^ In order to represent patients who would be likely to be referred for MPI, the inclusion criteria of this study were based on risk factors for coronary artery disease, as well as the presence of known coronary artery disease, whereas the ADVANCE trials recruited patients who were referred for clinically indicated MPI.

The findings of this study are consistent with the conclusions of previous pilot studies in subjects with asthma or COPD^5^,^6^ and indicate that the effect of regadenoson on the pulmonary function of these high-risk subjects is not clinically meaningfully different from placebo with respect to the percentage of subjects experiencing a >15% decrease in FEV_1_ from baseline to any assessment up to the 24-hour post-baseline follow-up visit. Although no subjects experienced severe bronchoconstriction in this study, the occurrence of these reactions cannot be ruled out with adenosine receptor agonists, such that the drug should be used with caution in patients with asthma and COPD, consistent with the prescribing information.^11^ This information should be useful when considering selection of regadenoson as a pharmacologic stress agent for MPI in these patient populations.
